# Coronavirus Disease 2019 (COVID-19)-Induced Takotsubo Cardiomyopathy Prognosis in Geriatric Setting

**DOI:** 10.7759/cureus.16211

**Published:** 2021-07-06

**Authors:** Mohsen S Alshamam, Nso Nso, Zarwa Idrees, Mahmoud Nassar, Most Sirajum Munira

**Affiliations:** 1 Internal Medicine, Icahn School of Medicine at Mount Sinai/New York City (NYC) Health+Hospitals Queens, Jamaica, USA; 2 Cardiology, Icahn School of Medicine at Mount Sinai/New York City (NYC) Health+Hospitals Queens, Jamaica, USA

**Keywords:** takotsubo cardioyopathy, stress-related cardiomyopathy, cardiogenic shock, reversible heart failure, cardiomyopathy

## Abstract

An 86-year-old female with a past medical history of hypertension, vertebral fractures with chronic lumbar pain, hip fracture, osteoporosis, deafness, and microcytic anemia underwent hospital admission for emergency medical management of her respiratory distress. The (overall) diagnostic workup confirmed COVID-19, the patient presented with 50% SPO2 (oxygen saturation), sinus tachycardia, diffuse bilateral pulmonary crackles, mild jugular venous distention (JVD), minimal bilateral pitting edema, elevated cardiac enzymes, bilateral pulmonary opacities, and ST-segment elevation. The cardiovascular assessment indicated stress-induced cardiomyopathy/Takotsubo cardiomyopathy (TCM) determined by 35%-40% LVEF (left ventricular ejection fraction), mid to apical left ventricular (LV) akinesia with preserved function in the proximal segment, aortic valve sclerosis, reduced excursion of Trileaflet valve (without stenosis), and mild-to-moderate tricuspid regurgitation with moderate pulmonary artery systolic pressure (PASP). The treatment protocol relied on 81 mg aspirin, 75 mg plavix, 20 mg lipitor, remdesivir, dexamethasone, ceftriaxone, azithromycin, red blood cells transfusion (pRBCs), endotracheal intubation for respiratory support, and systemic hemodynamic support. The patient’s condition did not improve despite all treatment, and she passed away after seven days following her hospital admission.

## Introduction

Takotsubo cardiomyopathy (TCM) is a stress-induced condition that leads to a transient, but reversible left ventricular dysfunction/wall motion abnormality without a reportable coronary obstruction [1]. The midventricular form of TCM follows its atypical nature. Studies did not find any gender difference observed in TCM with COVID-19 vs TCM without COVID-19 [2]. COVID-19 (coronavirus)-induced TCM is a recently reported condition that aggressively triggers cytokine storm with increased accumulation of catecholamines and cortisol. COVID-19 patients with TCM often experience a marked elevation in isometric tension, cardiomyocyte contraction, coronary spasm, and microvascular dysfunction. Patients also encounter a reduction in cardiac relaxation time and velocity. The ischemic episodes in TCM cases increase the risk of myocardial stunning that deteriorates the prognostic outcomes in the absence of reperfusion [3]. The cardiometabolic damage caused by COVID-19 infection in elderly patients substantially elevates their risk of cardiac deaths.

COVID-19 infection potentially increases the physical and socioemotional stress in the affected patients, increasing their TCM risk [4]. Studies also revealed an increasing trend of TCM development during the COVID-19 pandemic in non-covid or unexposed patients due to psychological stress or financial constraint [5]. Clinical decompensation due to COVID-19-induced TCM manifests through pulmonary hypertension and right ventricular dysfunction reflected by apical hypokinesis on transthoracic echocardiogram [6]. The septic/cardiogenic shock in the TCM setting is the primary cause of poor prognosis in COVID-19 infected elderly patients. The clinical management of TCM in the COVID-19 setting warrants advanced mechanical circulatory interventions supported by multidisciplinary teams. The respiratory decompensation followed by troponin elevation in COVID-19 scenarios potentially challenges the hospital course and therapeutic outcomes. Accordingly, vigilant treatment support and monitoring are conducive to modifying the prognostic outcomes in patients with COVID-19 induced TCM.

## Case presentation

**Figure 1:**
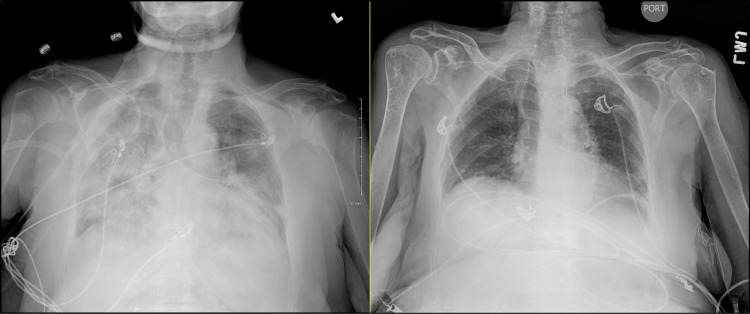
Chest X-ray (CXR) at admission (left) and a prior CXR for comparison (right). Diffuse bilateral pulmonary consolidation.

**Figure 2:**
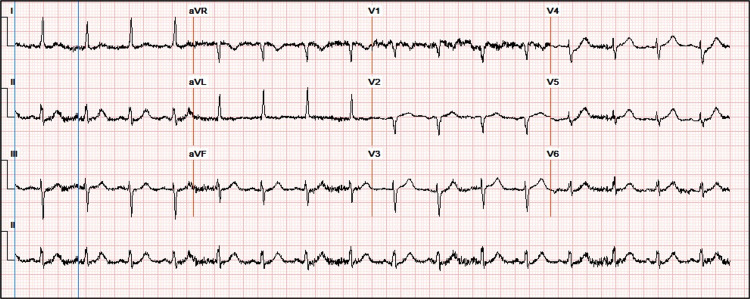
Electrocardiogram (ECG) at admission. No acute ischemic changes.

**Figure 3:**
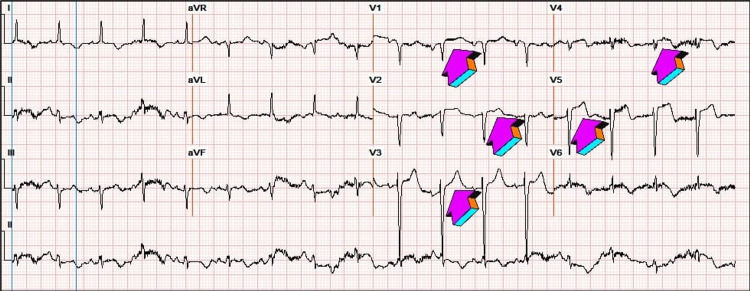
Repeat ECG one day after admission showing ST elevations in leads V1-V5.

**Figure 4:**
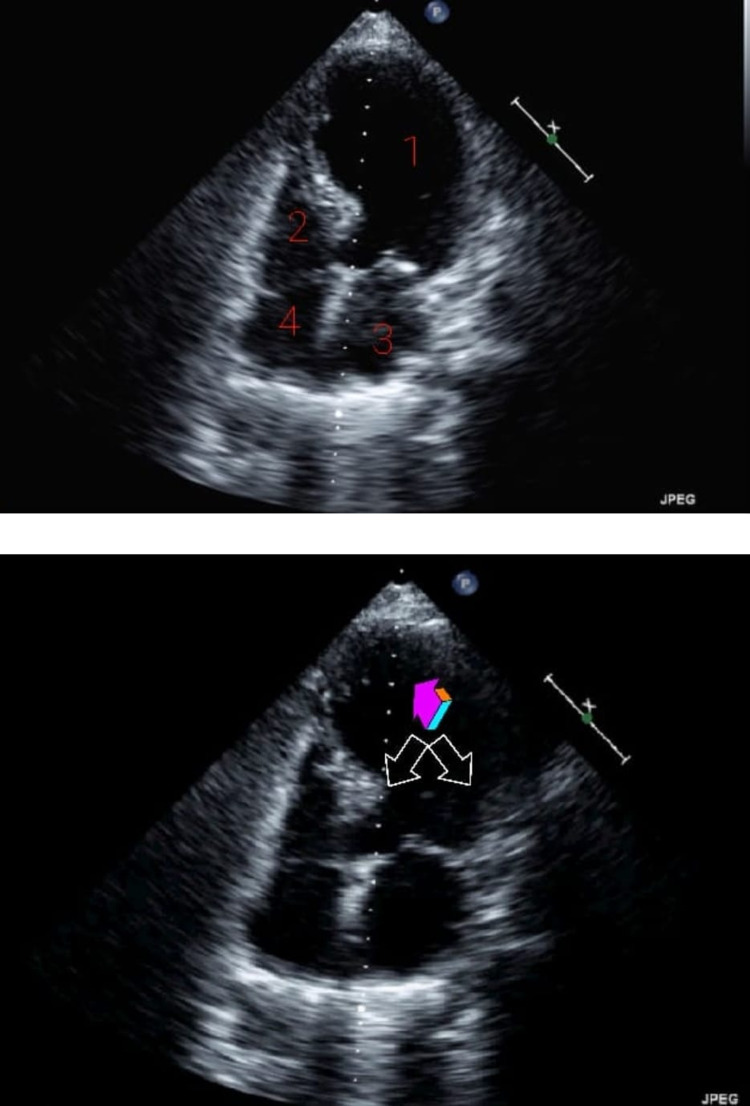
Echocardiogram (TTE) with four-chamber view of the heart. Top: 1. left ventricle (LV), 2. right ventricle (RV), 3. left atrium (LA), 4. right atrium (RA). Bottom: Single-colored arrow mid-apical segment ballooning. Double-arrows proximal/basal septum contracting inward.

An 86-year-old female was brought to the emergency room (ER) by emergency medical services (EMS) due to respiratory distress. The patients had a past medical history (PMH) of hypertension, vertebral fractures with chronic lumbar pain, hip fracture, osteoporosis, deafness, and microcytic anemia. The initial assessment revealed an SPO2 (oxygen saturation level) of 50% that improved to 80% via a non-rebreather mask with an oxygen flow rate capacity of 15 L/min. The respiratory deterioration of the patient warranted BiPAP (noninvasive bilevel positive airway pressure) intervention to achieve the SPO2 level of 95%. The assessment of vital signs revealed normal results, excluding the abnormal respiratory rate of 30 breaths per minute. The physical examination of the patient was consistent with respiratory distress, diffuse bilateral pulmonary crackles, mild jugular venous distention (JVD), and minimal bilateral pitting edema. The initial diagnostic workup revealed COVID-19 (coronavirus disease) and a marked elevation in cardiac enzymes/pro-B-type natriuretic peptides (Table 1 and Table 2). The chest X-ray findings were consistent with diffuse bilateral opacification attributing to pneumonia and/or pulmonary edema (Figure 1). The initial electrocardiogram (ECG) revealed normal sinus rhythm (NSR) in the absence of acute ischemic changes (Figure 2). A repeat ECG (at rest) on the second day of hospital admission confirmed sinus tachycardia determined by 101 beats per minute (BPM). The ECG findings also revealed ST-segment elevation in leads V1-V5 and T-wave inversions in leads I and aVL (Figure 3). A transthoracic echocardiogram (TTE) further indicated mid to apical left ventricular (LV) akinesia with preserved function in the proximal segment. The TTE images were typical for stress-induced cardiomyopathy/TCM (Figure 4). The findings also revealed 35%-40% LVEF (left ventricular ejection fraction), aortic valve sclerosis, reduced excursion of Trileaflet valve (without stenosis), and mild-to-moderate tricuspid regurgitation with moderate pulmonary artery systolic pressure (PASP). The cardiology consultation confirmed Takotsubo cardiomyopathy based on ECG/TTE changes and elevated cardiac enzymes. The cardiologist recommended continuous treatment with 81 mg aspirin, 75 mg plavix followed by the loading doses. The patient also received a heparin drip and 20 mg lipitor daily. The patient did not undergo left heart (cardiac) catheterization due to acute renal failure and the need for blood transfusion in the setting of severe microcytic anemia. The patient further required endotracheal intubation and systemic hemodynamic support to mitigate her clinical deterioration. The COVID-19 treatment protocol for the patient included dexamethasone and Remdesivir. The coadministration of antibiotics (ceftriaxone and azithromycin) relied on preventing secondary (bacterial) infection/sepsis. The patient’s condition, however, did not stabilize despite targeted therapy and life support interventions. The patient passed away following her continued decline after seven days of hospital admission.

**Table 1:** Troponin T initial and repeat according to the acute coronary syndrome (ACS) protocol.

Troponin T	Initial	Repeat	>>	>>	>>	>>	>>	>>	>>	>>	Reference Range (RR)
Value	0.016	0.334	0.384	0.59	0.756	0.813	1.18	1.51	1.53	1.18	=<0.010

**Table 2:** Initial lab values. *Cut-off value is the age of the patient x 5. pH: blood acidity; PCO2: partial pressure of carbon dioxide in the blood.

Lab Name	Value	Reference Range (RR)
Hemoglobin (Hgb)	5.3	12-16 g/dL
Hematocrit (Hct)	20.3	37-47%
White blood cells (WBC)	13.59	4.8-10.8 x10(3)/mcL
Platelets (Plt)	233	150-450x10(3)/mcL
Blood urea nitrogen (BUN)	24	6-23 mg/dL
Creatinine (Cr)	1.11	0.5-1.2 mg/dL
pH	7.119	7.32-7.42
PCO2	59.4	38-50 mmHg
Bicarbonate (HCO3)	18.4	22-28 mmol/L
Lactate	7.4	0.5-2.2 mmol/L
Pro B-Type Natriuretic Peptide (Pro-BNP)	2056	1-450 pg/mL
D-dimer	398	0-243 ng/mL DDU*
Procalcitonin (Pct)	0.09	0.02-0.10 ng/mL

## Discussion

The highest expression of COVID-19 is recorded in vascular endothelium and cardiomyocytes due to the activation of the angiotensin-converting enzyme-2 (ACE-2) receptor that possibly triggers myocardial injury [7]. The worsening of COVID-19-induced TCM prognosis is attributed to the elevated proinflammatory cytokines, which unprecedentedly elevate the cardiac load. The baseline characteristics of the coronavirus-infected TCM patients may also determine their prognostic deterioration. TCM with septic/cardiogenic shock further elevates the risk of cardiac death. These challenges advocate the need for advanced mechanical circulatory support to minimize the incidence of cardiovascular mortality in the setting of coronavirus-induced TCM. 

The potential causes of TCM in COVID-19 scenarios include catecholamine toxicity, microcirculatory dysfunction, epicardial spasm, and plaque rupture-induced transient ischemia [8]. The knowledge of pathological mechanisms dominating the prognosis of COVID-19 induced TCM is still in its infancy. Future studies should further investigate the concomitant role of intense emotional and physical stress (during the COVID-19 pandemic) in triggering TCM among predisposed patients. Cardiac catheterization (based on multiple EKG findings) followed by anticoagulants and diuretics include some of the preferred diagnostic and treatment options for TCM management in COVID-19 scenarios. 

The prognostic outcomes of COVID-19-induced TCM include thrombogenesis, arrhythmia, heart failure, left ventricular outflow tract obstruction, cardiogenic shock, and other lethal conditions [9]. It is important to predict the potential complications of TCM in COVID-19 emergencies to inform the treatment planning. The inclusion of coronary angiogram/left ventriculogram in diagnostic assessment may further help identify apical ballooning, basal hyperkinesis, and nonobstructive lesions in COVID-19 infected TCM patients [10].

## Conclusions

Future studies should focus on developing robust diagnostic algorithms for the early tracking of cardiac stress in COVID-19 patients. Personalized treatments relying on anticoagulation may require further modifications in concordance with the baseline characteristics and pathological pathways of TCM in COVID-19 scenarios.
